# Stable Longitudinal Screening of Latent Physiological Dysregulation from Psychometric Data Using Machine Learning

**DOI:** 10.3390/bioengineering13030339

**Published:** 2026-03-13

**Authors:** Alin Adrian Alecu

**Affiliations:** Faculty of Engineering in Foreign Languages (FILS), National University of Science and Technology Politehnica Bucharest, Splaiul Independentei 313, 060042 Bucharest, Romania; alin_adrian.alecu@upb.ro

**Keywords:** physiological dysregulation, psychometric screening, knowledge distillation, longitudinal risk prediction

## Abstract

Physiological dysregulation arising from chronic stress is a key mechanism linking psychosocial factors to long-term health outcomes, yet early identification typically relies on invasive or resource-intensive measurements. This study evaluates whether high-dimensional psychometric survey data can support scalable, non-invasive screening for latent physiological dysregulation. Using longitudinal data from the Midlife in the United States (MIDUS) Waves 2 and 3, we develop a screening-oriented modeling framework that separates longitudinal risk estimation from deployable screening model construction. Physiological targets are defined across inflammatory, metabolic, and neuroendocrine domains using three canonical allostatic load formulations. A teacher–ranking–pruning–student pipeline combines stable feature ranking, parsimony-driven dimensionality reduction, and knowledge distillation. Predictor dimensionality is reduced by more than an order of magnitude without loss of screening performance. Distilled student models consistently outperform linear, tree-based, and direct neural baselines, achieving area under the receiver operating characteristic curve values up to approximately 0.78 and substantial precision–recall lift over baseline prevalence. Longitudinal information is exploited during model development but not required at inference, enabling deployment using psychometric data alone. These findings demonstrate the feasibility of non-invasive screening for latent physiological dysregulation and provide a generalizable framework for translating longitudinal cohort data into deployable population health tools.

## 1. Introduction

Physiological dysregulation arising from chronic stress and cumulative life-course exposures is a central mechanism linking psychosocial factors to long-term health outcomes. The allostatic load framework formalizes this process as the progressive wear and tear across regulatory systems that results from repeated adaptation to environmental demands [1,2]. Dysregulation across inflammatory, metabolic, and neuroendocrine pathways has been associated with increased risk of cardiovascular disease, metabolic syndrome, cognitive decline, and mortality, making allostatic load a clinically meaningful but complex target for early identification and prevention [3,4].

Large population-based cohort studies have provided strong empirical support for these links. In particular, the Midlife in the United States (MIDUS) project has demonstrated systematic associations between psychosocial characteristics—including affect, personality traits, social relationships, and perceived stress—and biomarker-derived indices of physiological dysregulation [5]. These findings suggest that non-invasive psychometric data may contain actionable signal for identifying individuals at elevated biological risk, even in the absence of direct physiological measurements.

Despite this promise, translating such associations into deployable screening tools remains challenging. Psychometric instruments are typically high-dimensional, heterogeneous, and partially redundant, while cohort sample sizes are often modest relative to the number of available predictors. Naïve feature selection approaches in this regime are known to be unstable, undermining reproducibility and limiting clinical trust [6,7]. Moreover, while longitudinal cohort designs encode valuable temporal information, baseline data are rarely available at deployment, requiring careful separation between model development and inference-time requirements.

From an engineering perspective, screening for physiological risk therefore imposes a distinct set of constraints: models must handle extreme predictor dimensionality, exploit longitudinal structure during training, and ultimately yield compact, stable, and reproducible predictors suitable for large-scale deployment. These requirements are not well-addressed by purely cross-sectional models or by end-to-end learning approaches trained directly on high-dimensional survey inputs.

Recent advances in biomedical machine learning provide partial solutions to these challenges. In clinical informatics, temporally rich electronic health record data have been used to learn predictive representations that are subsequently compressed into static risk scores or deployable models [8,9]. In parallel, stability-aware feature selection methods have been proposed to improve robustness and interpretability in high-dimensional biomedical settings [6]. However, such approaches have rarely been applied to psychometric screening for latent physiological dysregulation, particularly when the final model must rely solely on non-invasive survey data.

Knowledge distillation offers a complementary mechanism for reconciling model capacity with deployability. Originally introduced to transfer information from complex models to simpler ones, distillation has been shown to preserve decision structure, improve generalization, and reduce variance in low-capacity models [10]. In biomedical applications, distillation has increasingly been used to translate complex predictive systems into models better suited for deployment, though applications to longitudinal screening tasks remain limited [11].

An additional challenge concerns the operationalization of physiological dysregulation itself. No single definition of allostatic load is universally accepted, and prior studies have employed a range of quantile-based, standardized, and clinically thresholded formulations [2,3,12,13]. As a result, conclusions drawn from a single target definition may reflect artifacts of the labeling scheme rather than robust physiological signal.

In this work, we address these challenges by developing a screening-oriented engineering framework for predicting latent physiological dysregulation from psychometric survey data. Using longitudinal data from MIDUS Waves 2 and 3, we design a teacher–ranking–pruning–student pipeline that explicitly separates high-capacity longitudinal risk estimation from compact, deployable screening model construction. By evaluating multiple canonical target definitions in parallel, we assess robustness to target operationalization while preserving strict non-circularity between predictors and physiological outcomes.

Our contributions are as follows:

- We introduce a longitudinal screening framework that transforms high-dimensional psychometric survey data into compact, deployable models for physiological risk screening.
- We demonstrate that stable feature ranking and parsimony-driven pruning can reduce predictor dimensionality by more than an order of magnitude without degrading longitudinal screening performance.
- We show that knowledge distillation enables low-capacity student models to preserve—and even improve upon—the discrimination performance of higher-capacity models across multiple physiological domains and target definitions.

Together, these results support the feasibility of scalable, non-invasive screening for latent physiological dysregulation using psychometric data alone, and provide a principled blueprint for translating longitudinal cohort information into deployable screening instruments.

## 2. Related Work

### 2.1. Allostatic Load and Physiological Dysregulation

The concept of allostatic load was introduced to characterize the cumulative physiological burden imposed by chronic stress and repeated regulatory demands across multiple biological systems. Early foundational work by McEwen and colleagues established allostatic load as a latent construct reflecting multisystem dysregulation rather than isolated biomarker abnormalities [1,14]. Subsequent epidemiological studies operationalized allostatic load using composite indices derived from panels of inflammatory, metabolic, and neuroendocrine biomarkers [2,15].

A key feature of the allostatic load literature is the absence of a single universally accepted operationalization. Prior studies have employed quantile-based thresholds, standardized (z-score) aggregations, and clinically defined cutoffs, often yielding different but overlapping risk profiles [3,13,16]. As a result, recent comparative studies highlight the importance of evaluating robustness across multiple target definitions rather than privileging a single scoring scheme [17].

Our work follows this established practice by evaluating quantile-based, z-score-based, and clinically thresholded targets in parallel, while holding biomarker composition fixed across definitions. This design isolates the impact of target construction from differences in biological measurement and aligns with canonical allostatic load methodology.

### 2.2. Psychosocial Determinants of Physiological Dysregulation in MIDUS

The Midlife in the United States (MIDUS) study has been widely used to examine associations between psychosocial factors and physiological dysregulation. A substantial body of work links stress exposure, affective and emotional regulation processes, and broader psychosocial characteristics to inflammatory and multisystem biological risk profiles using both cross-sectional and longitudinal analyses [18,19,20].

More recent MIDUS-based studies have moved beyond global associations to interrogate specific psychosocial pathways and life-course stressors that shape biological risk. Longitudinal analyses demonstrate that childhood maltreatment is associated with elevated allostatic load in midlife and older adulthood, while adult financial hardship predicts dysregulation in inflammatory biomarkers, underscoring the enduring physiological consequences of cumulative psychosocial adversity [19,21].

Beyond these pathways, several cohort studies have analyzed how specific psychological and social constructs predict trajectories of physiological dysregulation over time. Using MIDUS data, Brooks et al. [22] demonstrate that multiple dimensions of social relationships—including support, strain, and integration—are associated with longitudinal variation in allostatic load through statistical modeling approaches. In related work, Lewis and Hill [23] analyze data from the Health and Retirement Study (HRS) and the English Longitudinal Study of Ageing (ELSA) and show that psychological resources such as sense of purpose are associated with trajectories of physiological dysregulation across aging populations.

Taken together, these studies provide strong evidence that psychometric and psychosocial measures contain a meaningful signal related to multisystem biological risk. However, the dominant analytic paradigm in the literature is explanatory rather than predictive. Most studies focus on estimating associations, testing theoretical pathways, or characterizing population-level trajectories of physiological dysregulation using regression or multi-level modeling frameworks.

In contrast, relatively little work has addressed whether high-dimensional psychometric survey data can be leveraged to construct accurate and deployable screening models for latent physiological dysregulation. The present study complements the literature by shifting the emphasis from explanatory modeling to predictive screening. Rather than testing individual psychosocial pathways, we develop a machine learning framework designed to transform large sets of psychometric variables into compact models suitable for non-invasive population-level risk screening.

### 2.3. Predictive Screening and Risk Modeling from Behavioral Data

Machine learning approaches have been increasingly applied to health risk prediction using high-dimensional behavioral, survey, and electronic health record (EHR) data. Notable work by Miotto et al. introduced deep representation learning for patient risk stratification from EHRs, demonstrating that latent features can support accurate prediction across multiple clinical endpoints [8]. Goldstein et al. provided a comprehensive overview of machine learning methods for clinical risk prediction, emphasizing discrimination, calibration, and deployment considerations over causal interpretation [9].

Recent work has also explored other non-invasive behavioral modalities for predicting latent clinical states. For example, Vilenchik et al. [24] propose a voice-based framework for depression detection that combines acoustic features extracted from speech with a structured data collection protocol designed to improve label reliability and model generalization. Such studies demonstrate that behavioral signals, including voice, can provide predictive information about underlying clinical conditions and support scalable screening approaches based on non-invasive measurements.

A recurring theme in the literature is the use of longitudinal data to improve model training while maintaining cross-sectional inference at deployment. Temporal information is often leveraged during model development to learn stable risk representations that can subsequently be applied using static inputs [8,25]. This paradigm directly motivates our comparison between longitudinal teacher models and cross-sectional student models trained via distillation.

Related system-level work has articulated the clinical motivation for integrating behavioral and psychometric information into physiological risk assessment. For example, ref. [26] presents a conceptual framework discussing how non-invasive behavioral measures may complement biomarker-based assessment within broader clinical workflows. That work highlights the clinical relevance of behavioral screening and motivates the development of reliable, deployable modeling components capable of operating on high-dimensional psychometric data.

### 2.4. Feature Selection, Parsimony, and Deployability

High-dimensional predictor spaces pose challenges for interpretability, generalization, and deployment in biomedical settings. As a result, feature selection and dimensionality reduction have long been recognized as critical components of applied medical machine learning [27,28]. In screening contexts, parsimony is particularly important, as models must be robust to missing data, survey fatigue, and shifting data collection protocols.

Prior work has shown that reducing model complexity through feature selection or shrinkage can preserve or even improve predictive performance by mitigating overfitting and variance, particularly in moderate-sized cohorts [29,30]. Our parsimony analysis builds on the literature by explicitly quantifying the trade-off between dimensionality and screening performance across multiple physiological domains and target definitions.

### 2.5. Knowledge Distillation and Model Compression in Health Applications

Knowledge distillation was originally proposed as a framework for transferring the predictive behavior of high-capacity models into smaller, more efficient student models [10]. Subsequent work has extended distillation to multi-task settings and has demonstrated its utility for model compression, regularization, and improved generalization [31].

In biomedical machine learning, distillation has increasingly been used to translate computationally expensive predictive systems into models that are more suitable for clinical or population-level deployment. For example, teacher–student learning has been applied in clinical outcome prediction to transfer knowledge from high-capacity predictive models into more compact architectures that preserve much of the original model’s discrimination performance while substantially reducing computational complexity and inference cost [32].

Recent studies have also explored distillation strategies for settings in which the teacher model exploits temporally structured biomedical data. In such cases, the goal is often to compress information derived from longitudinal or time-series observations into predictive models that are easier to apply in practice. For instance, Fung et al. [33] introduced a self-knowledge distillation framework for microbiome time-series data, demonstrating that temporal predictive structure can be distilled into simplified models while preserving forecasting performance.

The present work adopts a related but distinct perspective. Rather than compressing time-series models themselves, we use knowledge distillation to transfer information learned from longitudinal cohort data into a screening model that operates entirely on cross-sectional inputs at inference time. In our framework, high-capacity teacher models leverage both baseline and follow-up psychometric predictors to estimate physiological risk, while the student model is trained to reproduce these probabilistic risk estimates using only the reduced set of follow-up survey variables available at deployment. This design enables temporal information present during model development to be encoded into a compact and deployable screening model that does not require longitudinal inputs at inference.

## 3. Materials and Methods

### 3.1. Data Source and Study Design

Data were obtained from the Midlife in the United States (MIDUS) study, a nationally representative longitudinal cohort designed to investigate psychosocial, behavioral, and biological determinants of health and aging. We used data from MIDUS Wave 2 (MIDUS 2) and Wave 3 (MIDUS 3), which include extensive psychometric survey assessments and objective biomarker measurements collected through dedicated Biomarker Projects. Throughout the remainder of the paper, these waves are denoted as M2 and M3, respectively.

Psychometric predictors were drawn exclusively from the MIDUS 2 and MIDUS 3 Survey Projects [34,35] and include self-report questionnaire items spanning psychological well-being, affect, personality traits, social relationships, stress exposure, health behaviors, and socioeconomic factors. These measures are non-invasive, low-burden, and broadly available, making them suitable for scalable screening applications.

Physiological supervision targets were derived exclusively from the MIDUS 2 and MIDUS 3 Biomarker Projects [36,37]. Biomarker variables were used solely to construct outcome labels and were never included as predictors, ensuring biological non-circularity.

All analyses follow a longitudinal screening paradigm. Psychometric survey data observed at baseline (MIDUS 2) and follow-up (MIDUS 3) are used to screen for elevated physiological dysregulation observed at follow-up. This design emphasizes temporal generalization and avoids optimistic bias associated with purely cross-sectional modeling.

### 3.2. Physiological Domains and Biomarker Composition

This study focuses on physiological dysregulation across multiple biological systems as conceptualized by the allostatic load framework, which characterizes the cumulative burden imposed by chronic stress and long-term regulatory imbalance.

We consider three physiological domains that are central to canonical allostatic load formulations: inflammatory (INFL), metabolic (META), and neuroendocrine (NEURO). Each domain is defined using a fixed set of biomarkers measured at both MIDUS waves. Domain-specific composite scores are later constructed from these biomarkers using alternative target definitions (described in the following subsection), but the underlying biomarker composition is held constant throughout all analyses.

Let $b_{i,j}^{\left(w\right)}$ denote the observed value of biomarker *j* for individual *i* at wave w∈{M2,M3}. For each physiological domain *d*, we define a corresponding biomarker set $B_{d}$ that specifies which biological measurements contribute to domain-level dysregulation.

#### 3.2.1. Inflammatory Domain (INFL)

The inflammatory domain is defined using the biomarker set

$$B_{\mathrm{INFL}}=\left\{\mathrm{CRP},\mathrm{IL}6,\mathrm{FGN}\right\},$$

where CRP denotes the C-reactive protein, IL6 denotes interleukin-6, and FGN denotes fibrinogen. All inflammatory biomarkers are treated as risk-increasing, such that higher values correspond to greater inflammatory dysregulation.

#### 3.2.2. Metabolic Domain (META)

The metabolic domain is defined using the biomarker set

$$B_{\mathrm{META}}=\left\{\mathrm{GLU},\mathrm{TG},\mathrm{HDL},\mathrm{SBP},\mathrm{DBP},\mathrm{WC}\right\},$$

where GLU denotes fasting glucose, TG triglycerides, HDL high-density lipoprotein cholesterol, SBP systolic blood pressure, DBP diastolic blood pressure, and WC waist circumference. HDL is treated as protective (risk-decreasing), whereas all other metabolic biomarkers are treated as risk-increasing.

#### 3.2.3. Neuroendocrine Domain (NEURO)

The neuroendocrine domain is defined using the biomarker set

$$B_{\mathrm{NEURO}}=\left\{\mathrm{COR},\mathrm{DHEA}\right\},$$

where COR denotes 24 h urinary cortisol and DHEA denotes dehydroepiandrosterone sulfate (DHEA-S). Cortisol is treated as risk-increasing, whereas DHEA-S is treated as protective (risk-decreasing).

Across all analyses, the biomarker composition of each domain is held fixed across MIDUS waves and across all target definitions. Consequently, differences between quantile-based, z-score-based, and clinically thresholded targets arise solely from how biomarker values are transformed and aggregated, rather than from changes in underlying biological measurement.

### 3.3. Target Definitions

Because no single operational definition of physiological dysregulation is universally accepted, we evaluate three canonical target constructions in parallel: quantile-based (Q), z-score-based (Z), and clinically thresholded (C). These definitions reflect common practices in the allostatic load literature and differ in their reliance on cohort-relative versus externally defined risk criteria. Evaluating all three allows us to assess robustness of screening performance to target construction and to avoid conclusions driven by a single labeling scheme.

For all definitions, targets are constructed separately for each physiological domain d∈{INFL,META,NEURO} and each MIDUS wave w∈{M2,M3}. Let $B_{d}$ denote the biomarker set defining domain *d*, and let $b_{i,j}^{\left(w\right)}$ denote the value of biomarker *j* for individual *i* at wave *w*.

#### 3.3.1. Quantile-Based Targets (Q)

For each biomarker *j*, a cohort-relative risk indicator is defined based on the empirical distribution of that biomarker. For risk-increasing biomarkers, individuals are assigned risk if their value lies in the upper quartile of the cohort distribution; for protective biomarkers, risk is assigned if the value lies in the lower quartile. Formally,

(1) $$r_{i,j}^{\left(Q,w\right)}=\left\{\begin{array}{cc} 1, & b_{i,j}^{\left(w\right)}\geq Q_{0.75}\left(b_{\;\cdot \;,j}^{\left(w\right)}\right)\;\left(\mathrm{risk}-\mathrm{increasing}\right),\\ 1, & b_{i,j}^{\left(w\right)}\leq Q_{0.25}\left(b_{\;\cdot \;,j}^{\left(w\right)}\right)\;\left(\mathrm{risk}-\mathrm{decreasing}\right),\\ 0, & \mathrm{otherwise}, \end{array}\right.$$

where $Q_{p}\left(\;\cdot \;\right)$ denotes the *p*th empirical quantile.

The domain-specific quantile score is then

(2) $$S_{i,d}^{\left(Q,w\right)}=\sum_{j\in B_{d}}r_{i,j}^{\left(Q,w\right)}.$$

Binary high-risk labels are assigned as

(3) $$y_{i,d}^{\left(Q,w\right)}=I\;\left(S_{i,d}^{\left(Q,w\right)}\geq \left\lceil \frac{|B_{d}|}{2}\right\rceil \right),$$

corresponding to elevated risk in at least half of the domain’s biomarkers. Here, I(·) denotes the indicator function which takes the value 1 if the condition inside the parentheses is satisfied and 0 otherwise, while $|B_{d}|$ denotes the cardinality of the biomarker set defining domain *d*.

#### 3.3.2. Z-Score-Based Targets (Z)

Each biomarker is standardized within the analytic cohort at each wave,

(4) $$z_{i,j}^{\left(w\right)}=\frac{b_{i,j}^{\left(w\right)}-\mu_{j}^{\left(w\right)}}{\sigma_{j}^{\left(w\right)}},$$

where $\mu_{j}^{\left(w\right)}$ and $\sigma_{j}^{\left(w\right)}$ denote the mean and standard deviation of biomarker *j* at wave *w*. Protective biomarkers are sign-reversed so that higher values consistently correspond to greater dysregulation.

Domain-specific z-score composites are defined as

(5) $$S_{i,d}^{\left(Z,w\right)}=\frac{1}{|B_{d}|}\sum_{j\in B_{d}}z_{i,j}^{\left(w\right)}.$$

Binary high-risk labels are assigned by thresholding the composite score at the upper quartile of its empirical distribution,

(6) $$y_{i,d}^{\left(Z,w\right)}=I\;\left(S_{i,d}^{\left(Z,w\right)}\geq Q_{0.75}\left(S_{\;\cdot \;,d}^{\left(Z,w\right)}\right)\right).$$

This approach follows the standard practices for constructing composite physiological risk scores, with statistical considerations for sample size and distributional properties discussed in [38].

#### 3.3.3. Clinically Thresholded Targets (C)

For biomarkers with established clinical reference ranges, binary risk indicators are defined using externally specified thresholds derived from clinical guidelines or MIDUS conventions,

(7) $$r_{i,j}^{\left(C,w\right)}=\left\{\begin{array}{cc} 1, & b_{i,j}^{\left(w\right)}\in \mathrm{clinically}\;\mathrm{high}-\mathrm{risk}\;\mathrm{range},\\ 0, & \mathrm{otherwise}. \end{array}\right.$$

The clinical composite score is

(8) $$S_{i,d}^{\left(C,w\right)}=\sum_{j\in B_{d}}r_{i,j}^{\left(C,w\right)},$$

and high-risk labels are assigned using the same majority rule,

(9) $$y_{i,d}^{\left(C,w\right)}=I\;\left(S_{i,d}^{\left(C,w\right)}\geq \left\lceil \frac{|B_{d}|}{2}\right\rceil \right).$$

Table 1 lists the biomarker-specific clinical cutoffs used to define high-risk values under the clinical (C) target definition. These thresholds are applied uniformly across MIDUS waves and are domain-specific where relevant.

Across all definitions, targets are computed independently at MIDUS 2 and MIDUS 3, yielding longitudinally aligned binary outcomes that serve as supervision targets for the screening models.

### 3.4. Predictor Universe Construction

To ensure biological non-circularity, longitudinal consistency, and deployability, candidate predictors were restricted to psychometric survey variables collected as part of the MIDUS Survey Projects. All predictors are non-invasive, self-reported measures and are distinct from the biomarker variables used to construct the physiological targets.

Only survey variables with valid counterparts in both MIDUS 2 (baseline) and MIDUS 3 (follow-up) were retained, enforcing cross-wave availability and ensuring that the same predictor definitions are applicable under longitudinal screening. This constraint is critical for both model training and downstream deployment, where predictor availability must be stable over time.

A series of deterministic exclusion rules was applied to remove variables that are either biologically inappropriate, administratively defined, or unsuitable for screening applications. Specifically, all biomarker variables, derived allostatic load components, biospecimen collection metadata, participant identifiers, linkage fields, sampling weights, and process-related variables were excluded. Redundant or alternative questionnaire variants were collapsed using canonical MIDUS conventions to avoid duplicate representations of the same construct.

Finally, predictors that contained no observed values or exhibited zero variance after numeric coercion were removed, ensuring that all retained variables contribute usable information in the analytic sample.

After applying all inclusion and exclusion criteria, the final predictor universe contained p=1904 psychometric survey variables. The full set of filtering rules, together with their operational definitions and rationale, is summarized in Table 2.

### 3.5. Problem Formulation and Screening Objective

The objective of this study is to design and evaluate a screening model for latent physiological dysregulation using non-invasive psychometric survey data. The task is formulated as a supervised binary classification problem under a longitudinal prediction setting.

For each individual *i* and physiological domain d∈{INFL,META,NEURO}, the goal is to predict whether the individual exhibits elevated physiological dysregulation at follow-up (MIDUS 3). Let $y_{i,d}^{M3}\in \left\{0,1\right\}$ denote the corresponding domain-specific high-risk label derived from biomarker data using one of the target definitions described in Section 3.3. A value of $y_{i,d}^{M3}=1$ indicates elevated dysregulation in domain *d* at follow-up.

Model inputs consist exclusively of psychometric survey responses. Let $X_{i}^{M3}\in R^{p}$ denote the vector of follow-up survey predictors for individual *i*, restricted to variables with valid counterparts at both MIDUS 2 and MIDUS 3. Although longitudinal information is exploited during training, all models are evaluated under a cross-sectional inference constraint: at deployment time, predictions are generated using only $X_{i}^{M3}$, without access to baseline survey data or biomarker measurements.

Accordingly, the screening function for each domain can be written as

(10) $$f_{d}:X_{i}^{M3}\mapsto {\hat{p}}_{i,d},$$

where ${\hat{p}}_{i,d}\in \left[0,1\right]$ denotes the model-produced screening probability that individual *i* exhibits elevated physiological dysregulation in domain *d* at follow-up. This probability represents the final output of the screening model and is the quantity used for all performance evaluation. Binary screening decisions may be obtained by thresholding ${\hat{p}}_{i,d}$, but all reported results are based on threshold-independent metrics.

This formulation emphasizes early identification of individuals at elevated physiological risk rather than clinical diagnosis. Biomarker data are used solely to construct supervision targets and are never available at inference, ensuring that the resulting models are deployable in large-scale, low-burden screening settings where biological sampling is impractical or unavailable.

### 3.6. Modeling and Evaluation Framework

The modeling strategy is designed to support a screening-oriented engineering objective rather than purely maximizing predictive accuracy. Specifically, the framework aims to (i) learn robust longitudinal risk representations from high-dimensional psychometric data, (ii) identify a compact and stable subset of predictors suitable for deployment, and (iii) distill longitudinal information into a deployable screening model that operates under cross-sectional inference constraints.

**Figure 1:**
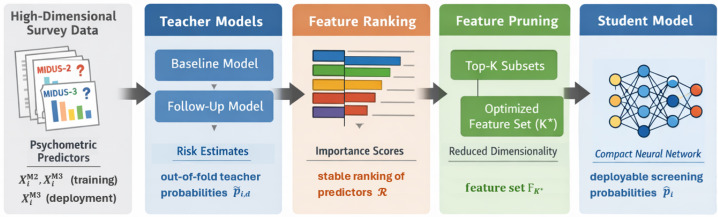
Overview of the screening-oriented modeling pipeline. Psychometric survey predictors collected at MIDUS Wave 2 (baseline) and Wave 3 (follow-up) are used to train high-capacity longitudinal teacher models for each physiological domain (inflammatory, metabolic, and neuroendocrine). Teacher models produce domain-specific follow-up risk probabilities, which are used both to derive stable feature importance rankings and to provide probabilistic supervision for downstream models. Aggregated rankings are used to select an optimized reduced predictor set under a tolerance-based criterion. A compact student model is then trained via knowledge distillation on the pruned feature set, yielding a deployable screening model for latent physiological dysregulation.

To meet these goals, we adopt a staged framework consisting of teacher risk estimation, stable feature ranking, parsimony-based feature pruning, and student model distillation. An overview of the pipeline is shown in Figure 1.

#### 3.6.1. Longitudinal Teacher Models

Teacher models serve as high-capacity estimators of follow-up physiological risk and are used to extract both probabilistic supervision signals and stable feature importance rankings. For each physiological domain d∈{INFL,META,NEURO} and each target definition (Q, C, Z), teacher models are trained under a two-stage longitudinal design.

In the first stage, the baseline survey predictors $X_{i}^{M2}$ are used to estimate baseline risk. In the second stage, the follow-up predictors $X_{i}^{M3}$ are augmented with the baseline-derived risk estimate to predict follow-up risk. This structure allows longitudinal information to influence training while preserving a cross-sectional inference interface.

In the first stage, baseline survey predictors $X_{i}^{M2}\in R^{p}$ are used to estimate a baseline-stage risk probability,

(11) $${\hat{p}}_{i,d}^{M2}=g_{d}\;\left(X_{i}^{M2}\right),$$

where $g_{d}\left(\;\cdot \;\right)$ denotes a domain-specific baseline risk estimator.

In the second stage, follow-up survey predictors $X_{i}^{M3}$ are combined with the baseline-stage estimate ${\hat{p}}_{i,d}^{M2}$ to predict follow-up risk. Specifically, the teacher model produces an out-of-fold probability estimate

(12) $${\tilde{p}}_{i,d}=f_{d}^{\mathrm{RF}}\;\left(X_{i}^{M3},\;{\hat{p}}_{i,d}^{M2}\right),$$

where $f_{d}^{\mathrm{RF}}\left(\;\cdot \;\right)$ denotes a domain-specific random forest classifier. The quantity ${\tilde{p}}_{i,d}\in \left[0,1\right]$ represents the teacher’s estimate of the probability that individual *i* exhibits elevated physiological dysregulation in domain *d* at follow-up.

Random forests are used as teacher models due to their ability to capture nonlinear interactions, accommodate mixed-scale predictors, and remain robust under multicollinearity and high-dimensional input spaces. All teacher outputs ${\tilde{p}}_{i,d}$ and baseline-stage estimates ${\hat{p}}_{i,d}^{M2}$ used downstream are obtained strictly from out-of-fold predictions to prevent information leakage.

Importantly, the teacher probabilities ${\tilde{p}}_{i,d}$ are used only during training to (i) derive stable feature rankings and (ii) provide soft supervision signals for student model distillation. They are never used at inference time. This design ensures that longitudinal information influences model learning while preserving a purely cross-sectional screening interface for deployment.

#### 3.6.2. Stable Feature Ranking

Direct feature selection from a single fitted model is known to be unstable in high-dimensional biomedical settings. Recent work on feature selection benchmarking further emphasizes that many commonly used datasets are not inherently challenging and argues for explicit hardness criteria when evaluating feature selection methods, with difficulty linked to the utility gained from feature selection and related data characteristics [39].

To obtain robust predictor rankings, feature importance scores are extracted from the outcome-stage (MIDUS 3) teacher models separately within each cross-validation fold.

Fold-specific importance scores are then aggregated across folds to produce a stable global ranking of psychometric predictors for each physiological domain. This aggregation emphasizes predictors that contribute consistently to longitudinal risk estimation rather than those selected due to sampling variability.

Feature ranking is performed independently for each domain and target definition, ensuring that domain-specific screening signals are preserved.

#### 3.6.3. Parsimony-Based Feature Pruning

Using the aggregated rankings, nested subsets of the top-*K* predictors are constructed for *K* ranging from small (highly compact) to near-full representations. For each subset, teacher models are retrained and evaluated using identical out-of-fold protocols.

Rather than selecting *K* to maximize performance, we adopt a tolerance-based parsimony criterion. Let AUC(K) denote the mean out-of-fold area under the receiver operating characteristic curve, averaged across domains for a given *K*. The selected feature set size $K^{*}$ is defined as the smallest *K* satisfying

(13) $$\mathrm{AUC}\left(K\right)\geq \max_{K^{\prime }}\mathrm{AUC}\left(K^{\prime }\right)-\epsilon ,$$

where ϵ is a fixed tolerance. This criterion explicitly prioritizes dimensionality reduction and stability over marginal accuracy gains.

The resulting parsimony-optimal feature sets ($K_{Q}^{*}$, $K_{C}^{*}$, $K_{Z}^{*}$) form the basis for all subsequent model comparisons.

#### 3.6.4. Student Models and Knowledge Distillation

Although teacher models provide strong performance, they are not well-suited for deployment due to their size and complexity. To obtain a compact and deployable screening model, we train a low-capacity neural network as a student model using knowledge distillation.

The student model takes as input only the pruned follow-up predictors $X_{i,K^{*}}^{M3}\in R^{K^{*}}$ and jointly predicts the three domain-specific risks. Let

(14) $${\hat{p}}_{i}=\left({\hat{p}}_{i,\mathrm{INFL}},{\hat{p}}_{i,\mathrm{META}},{\hat{p}}_{i,\mathrm{NEURO}}\right)$$

denote the student’s output.

Training combines supervision from the observed binary follow-up labels $y_{i,d}^{M3}$ and imitation of the probabilistic outputs ${\tilde{p}}_{i,d}$ produced by the longitudinal teacher models. The student network is trained using a composite distillation loss,

(15) $$L_{\mathrm{distill}}=\sum_{d}\left[\lambda \;\mathrm{BCE}\;\left(y_{i,d}^{M3},{\hat{p}}_{i,d}\right)+\left(1-\lambda \right)\;{∥{\hat{p}}_{i,d}-{\tilde{p}}_{i,d}∥}^{2}\right],$$

where BCE(·,·) denotes the binary cross-entropy loss, which encourages accurate prediction of the observed high-risk labels. The second term penalizes deviations between the student predictions ${\hat{p}}_{i,d}$ and the teacher probabilities ${\tilde{p}}_{i,d}$, thereby transferring longitudinal structure learned by the teacher into the student model. The mixing parameter λ∈(0,1) controls the trade-off between direct label supervision and fidelity to the teacher decision function.

During training, the composite distillation loss decreases monotonically until convergence, indicating stable optimization of the student model. Because the focus of this study is screening performance rather than training dynamics, model evaluation is based on out-of-fold predictive performance rather than training loss trajectories.

This distillation step allows longitudinal information learned by the teachers to be compressed into a compact model that operates solely on cross-sectional survey inputs at inference time.

The main training hyperparameters used for the teacher and student models are summarized in Table 3.

#### 3.6.5. Evaluation Protocol and Metrics

All models are evaluated using stratified *k*-fold cross-validation with k=5. The same data splits are used for all models to ensure fair comparison, and all reported performance metrics are computed from out-of-fold predictions. Because each prediction is generated by a model that was not trained on the corresponding observation, this protocol provides an unbiased estimate of generalization performance and mitigates overfitting during model evaluation.

Screening performance is assessed using the area under the receiver operating characteristic curve (AUC) and average precision (AP). AUC quantifies overall discrimination ability, while AP is sensitive to class imbalance and is particularly relevant in screening contexts where positive cases may be rare.

Unless otherwise stated, all reported results correspond to follow-up (MIDUS 3) screening performance using only follow-up psychometric predictors at inference time.

## 4. Results

### 4.1. Cohort, Task Definition, and Class Balance

All results are reported for the longitudinal screening task defined in Section 3.5. Psychometric survey predictors collected at MIDUS Wave 2 (baseline) and Wave 3 (follow-up) were used to screen for elevated physiological dysregulation observed at follow-up. After enforcing cross-wave availability (requiring valid MIDUS 2 and MIDUS 3 counterparts) and applying all deterministic predictor eligibility filters (Table 2), the candidate predictor universe consisted of p=1904 survey variables.

The analytic cohort comprised n=584 individuals with complete linkage across MIDUS 2 and MIDUS 3 for both survey predictors and biomarker-derived targets. Screening targets were defined for three physiological domains—inflammatory (INFL), metabolic (META), and neuroendocrine (NEURO)—under three alternative target constructions: quantile-based (Q), clinical threshold-based (C), and z-score-based (Z), as described in Section 3.3. MIDUS-3 positive class counts and other metrics are shown in Table 4, where it can be seen that class prevalence varies substantially across target definitions and domains, motivating the joint use of AUC and AP as primary screening metrics throughout this section.

### 4.2. Feature Parsimony and Dimensionality Reduction

**Figure 2:**
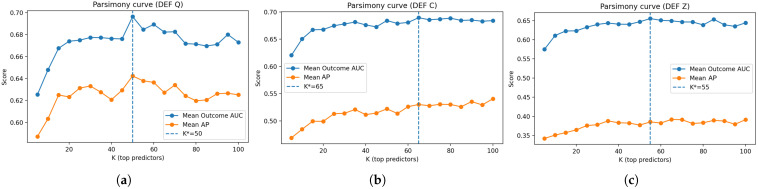
Parsimony analysis of longitudinal screening models under three physiological target definitions: (**a**) quantile-based (Q), (**b**) clinically thresholded (C), and (**c**) z-score-based (Z). Each panel reports mean out-of-fold outcome AUC and average precision (AP), averaged across inflammatory, metabolic, and neuroendocrine domains, as a function of the number of retained psychometric predictors. Vertical dashed lines indicate the selected parsimony-optimal feature set size $K^{*}$.

We first evaluated the extent to which high-dimensional psychometric predictors could be compressed without degrading longitudinal screening performance. Following stable feature ranking derived from longitudinal teacher models, nested subsets of the top-*K* predictors were evaluated for K∈{5,…,100} under identical out-of-fold (OOF) evaluation protocols, the parsimony curves for the three target definitions being depicted in Figure 2.

Across all target definitions, it can be seen from Figure 2 that screening performance improves rapidly as *K* increases from very small values, followed by a broad plateau in which additional predictors yield diminishing returns. Performance remains stable across wide intermediate ranges of *K*, indicating that predictive signal is concentrated in a relatively small subset of psychometric variables rather than being diffusely distributed across the full survey space.

Using a tolerance-based criterion that prioritizes minimal dimensionality subject to near-optimal performance, parsimony-optimal feature set sizes were selected as $K_{Q}^{*}=50$, $K_{C}^{*}=65$, and $K_{Z}^{*}=55$. In all cases, this corresponds to a reduction of more than an order of magnitude relative to the original predictor space. These reduced feature sets are used in all subsequent model comparisons.

To quantify the effect of dimensionality reduction, we compared in Table 5 the outcome-stage (MIDUS 3) performance of longitudinal random forest teacher models trained on the full predictor set (FULL) and on the parsimony-optimal subset ($K^{*}$). The model architecture and evaluation protocol were held fixed.

It can be seen that across all target definitions and physiological domains, aggressive dimensionality reduction from 1904 predictors to the parsimony-optimal subsets yields consistent improvements in both AUC and average precision. Importantly, no degradation in screening performance is observed in any setting. These results indicate that pruning to the optimized feature sets not only preserves longitudinal predictive signal but also reduces noise and redundancy, demonstrating that the signal is concentrated in a compact subset of predictors and that performance gains are robust to target definition and physiological domain.

### 4.3. Model Performance on Parsimony-Optimal Feature Sets

We next compared alternative modeling strategies using only the parsimony-optimal predictor sets identified in Section 4.2 ($K_{Q}^{*}=50$, $K_{C}^{*}=65$, $K_{Z}^{*}=55$). All results are based on out-of-fold (OOF) predictions obtained under identical longitudinal data splits, ensuring that observed performance differences reflect modeling choices rather than data partitioning.

The purpose of this comparison is to benchmark a deployable screening model that operates at inference time using a compact set of follow-up psychometric predictors $X^{M3}\in R^{n\times K^{*}}$, while evaluating whether longitudinal structure available during training can be transferred into such a cross-sectional model. Accordingly, all models discussed herein output continuous risk scores ${\hat{s}}_{i,d}\in \left[0,1\right]$ for each physiological domain d∈{INFL,META,NEURO}, interpreted as predicted probabilities of follow-up high-risk status. Binary decisions are obtained only through post hoc thresholding, whereas AUC and AP are computed from the continuous scores.

We evaluated a spectrum of model classes commonly used in screening and risk prediction, spanning increasing representational capacity. Random forests trained independently for each domain using only MIDUS 3 predictors (denoted “RF M3-only”) serve as strong nonlinear baselines without explicit longitudinal supervision. Ridge-regularized logistic regression (denoted “Logit L2”) and elastic net (denoted “Elastic Net”) models provide linear reference points under the same feature constraints. A compact multi-output neural network (denoted “MLP direct”), trained end-to-end on the pruned predictors, represents a flexible yet low-capacity neural baseline capable of modeling shared structure across domains.

To assess the added value of longitudinal supervision without increasing inference-time data requirements, we also evaluated a distilled student neural network (denoted “MLP distill”) corresponding exactly to the student model described in Section 3.6. This model uses the same architecture and the same follow-up predictors $X^{M3}$ as the direct MLP, but is trained using a combined objective that incorporates both the observed binary labels and the out-of-fold probability outputs produced by longitudinal teacher models. Importantly, these teacher-derived probabilities are used only during training; the distilled student does not require baseline (MIDUS 2) information at deployment. This design allows us to test whether temporal information can be compressed into a deployable screening model that remains purely cross-sectional at inference. The performance comparison is depicted in Table 6.

Several consistent patterns emerge from Table 6. Across target definitions and physiological domains, nonlinear models outperform linear baselines, confirming that even after aggressive dimensionality reduction, meaningful nonlinear structure remains in the psychometric predictor space. Random forests provide a strong classical reference, while the direct multi-output MLP achieves competitive but less consistent performance, particularly in domains with weaker signal separation.

The distilled student model (MLP distill) achieves the strongest and most consistent performance across all domains and target definitions. By incorporating longitudinal teacher supervision during training while remaining fully cross-sectional at inference, the distilled MLP consistently exceeds the performance of both classical baselines and direct neural training. These gains are observed for all three target constructions, indicating that the benefits of distillation are robust to how physiological dysregulation is operationalized.

We reported the area under the receiver operating characteristic curve (AUC) as a primary measure of discrimination. In population-level screening and behavioral risk prediction tasks—particularly when outcomes are multifactorial, proxy-based, or derived from composite physiological indices—AUC values in the range of approximately 0.70–0.80 are widely reported in prior work and regarded as practically useful for risk stratification and downstream triage rather than definitive diagnosis [8,9]. Within this context, the distilled student models achieve AUC values up to approximately 0.78 (metabolic dysregulation under the quantile-based definition), with consistent gains over linear, tree-based, and direct neural baselines across physiological domains and target constructions. These values place the proposed approach squarely within the range reported in prior work on non-invasive and behavioral screening using longitudinal or high-dimensional inputs.

Because all tasks considered here exhibit substantial class imbalance (Table 4), we additionally reported average precision (AP), equivalent to the area under the precision–recall curve. As established in the methodological literature, AP must be interpreted relative to outcome prevalence: for a non-informative (random) ranking, expected AP equals the positive class rate, so absolute AP values are not directly comparable across tasks with different prevalences [40,41]. Accordingly, AP is most informative when expressed relative to this baseline.

To facilitate such interpretation, Table 7 reports AP lift, defined as the ratio between observed AP and class prevalence, for the best-performing distilled student model. AP lift directly quantifies the degree to which true high-risk individuals are enriched among top-ranked predictions relative to chance.

Across all settings, AP lift ranges from approximately 1.3× to over 2.1× relative to a random ranking, indicating substantial enrichment of high-risk individuals among the highest-ranked predictions. Lift values of this magnitude are consistent with, and in several cases exceed, those reported in prior clinical and behavioral screening studies operating under comparable class imbalance, where performance is evaluated by enrichment of true positives among the top-ranked individuals rather than diagnostic accuracy. For example, prior work on imbalanced health risk stratification tasks reports cumulative lift values on the order of 1.5×–2.0× in top-ranked subsets when predicting adverse clinical outcomes from high-dimensional observational data [42,43]. Taken together with the observed AUC values, these results indicate that the distilled student models provide competitive and clinically relevant screening performance, particularly given their exclusive reliance on non-invasive psychometric predictors and the strict separation between training-time longitudinal information and deployment-time inputs.

### 4.4. Interpretation of Selected Predictors

While the feature selection pipeline ensures stability and parsimony of the predictor set, interpreting the retained variables is essential for understanding the behavioral and clinical relevance of the learned models. To this end, we examine the predictors with the highest permutation importance and analyze their distribution across conceptual domains.

Table 8 reports the most influential predictors ranked by their mean permutation importance, measured as the decrease in model AUC (Δ AUC) after randomly permuting each predictor. Importance values are aggregated across nine model variants corresponding to three dysregulation definitions (Q, C, Z) and three physiological subsystems (inflammatory, metabolic, neuroendocrine). Because physiological dysregulation arises from multiple correlated behavioral and biological risk factors, individual predictors typically produce modest decreases in AUC while collectively capturing a substantial predictive signal. The Q, C, and Z columns indicate the specific model variants in which the predictor contributes non-zero importance, allowing assessment of cross-model stability.

The retained predictors span multiple domains including anthropometric indicators, health behaviors, socioeconomic characteristics, and psychosocial constructs measured through survey instruments.

Several patterns emerge from these results. First, anthropometric and cardiometabolic indicators constitute the most influential predictors in terms of individual importance. Body mass index, waist circumference, hip circumference, and hypertension-related variables consistently appear among the top-ranked predictors across multiple model variants. This pattern suggests that metabolic and cardiovascular risk factors capture a substantial portion of the variance in physiological dysregulation within the MIDUS cohort, consistent with prior work showing that metabolic components often contribute strongly to composite allostatic load indices.

Second, socioeconomic indicators such as income, financial assets, and employment history also contribute a predictive signal. Variables such as the respondent’s wages, IRA assets, and years of employment appear across multiple models, suggesting that socioeconomic position captures long-term stress exposure and cumulative disadvantage that influence physiological dysregulation.

Third, the results highlight the importance of psychosocial constructs. Although psychological variables do not dominate the highest-ranked predictors individually, they appear frequently in the retained feature set. Table 9 summarizes the distribution of retained variables across conceptual domains.

Notably, psychological and social variables constitute the largest domain among the retained predictors, accounting for approximately one third of the feature set. When combined with socioeconomic and health behavior variables, behavioral and psychosocial factors represent approximately 58% of all retained predictors. This pattern suggests that psychosocial factors contribute predictive information through multiple moderately informative variables rather than a small number of dominant predictors.

These findings are consistent with prior research examining psychosocial determinants of physiological dysregulation. In the MIDUS cohort, Brooks et al. [22] showed that multiple dimensions of social relationships—including social integration, emotional support, and relationship strain—are associated with levels of allostatic load across cardiovascular, metabolic, and inflammatory systems. Individuals reporting greater relationship strain exhibited higher allostatic load scores, whereas higher levels of emotional support were associated with lower physiological dysregulation. Consistent with these findings, relationship-related constructs such as the “Positive relations with others” scale from the psychological well-being inventory appear among the predictors contributing to inflammatory and neuroendocrine dysregulation in our models.

Similarly, Lewis and Hill [23] examined the association between psychological purpose and physiological regulation using longitudinal data from the Health and Retirement Study and the English Longitudinal Study of Ageing. Their analyses showed that individuals with higher levels of purpose in life exhibited lower overall levels of allostatic load across repeated biomarker assessments, although purpose did not significantly influence the rate of change in allostatic load over time. Our results align with this interpretation, as psychological well-being indicators such as subjective age (“Age feel like most of the time”) appear among the retained predictors. Subjective age has been shown in prior research to correlate with health outcomes and biological aging markers, suggesting that individuals’ perceptions of their own aging may reflect underlying physiological processes.

Another notable pattern is the stability of several predictors across dysregulation definitions and physiological subsystems. Variables such as body mass index and waist circumference exhibit non-zero importance across nearly all model variants, indicating that they capture signals that generalize across different operationalizations of physiological dysregulation. Such stability suggests that the feature selection pipeline identifies predictors reflecting general physiological risk rather than signals specific to a particular dysregulation definition.

Importantly, the predictors identified by the feature selection pipeline are not themselves components of the biomarker-based dysregulation indices. Instead, they represent upstream behavioral, socioeconomic, and psychosocial factors associated with physiological risk. This suggests that the model captures determinants of physiological dysregulation rather than merely reconstructing the biomarker definitions used to compute the outcome variables.

Taken together, the retained predictors form three conceptual layers: direct physiological indicators reflecting current biological risk, socioeconomic variables capturing long-term stress exposure, and psychosocial variables representing relational and psychological resources. The feature selection results therefore reveal a coherent multi-domain structure in which physiological indicators capture current biological risk, while socioeconomic and psychosocial variables reflect upstream determinants of physiological dysregulation.

## 5. Discussion

This study addresses a practical challenge at the interface of computational modeling and population health: how to construct deployable, non-invasive screening models for latent physiological dysregulation from high-dimensional psychometric data. Rather than optimizing a single predictive model, we focused on designing an engineering pipeline that reconciles competing constraints—dimensionality, longitudinal structure, robustness, and deployability—commonly encountered in large-scale cohort studies.

To position the present work within the broader literature, Table 10 summarizes the representative studies examining psychosocial determinants of physiological dysregulation and related behavioral health prediction tasks. Prior work has primarily focused on explanatory analyses that examine statistical associations between psychosocial variables and biomarker-derived indices of allostatic load, typically using regression-based modeling frameworks. In contrast, the present study adopts a predictive screening perspective, aiming to transform high-dimensional psychometric survey data into deployable models capable of identifying individuals at elevated physiological risk.

Prior MIDUS-based studies have primarily focused on examining associations between psychosocial factors and physiological dysregulation rather than developing predictive screening models. Consequently, these studies typically report regression coefficients, effect sizes, or statistical significance rather than discrimination metrics such as the area under the receiver operating characteristic curve (AUC). For this reason, direct numerical comparison of predictive performance with prior MIDUS analyses is not appropriate. Instead, the comparison is conceptual: the predictors identified by the machine learning pipeline in this work—such as anthropometric indicators, socioeconomic variables, and psychosocial resources—closely align with determinants of physiological dysregulation previously reported in epidemiological research.

A central finding of this study is that robust longitudinal screening signal is concentrated in a relatively small subset of psychometric variables. Across all three target definitions, aggressive dimensionality reduction from approximately 1900 survey predictors to compact sets of 50–65 variables not only preserved discrimination performance but consistently improved it. These gains indicate that psychometric survey data contain substantial structured redundancy, and that principled feature ranking and pruning can amplify a signal by reducing noise and variance. From a deployment perspective, this level of parsimony is critical, as it substantially lowers data collection burden, enhances stability across samples, and enables the construction of reproducible and scalable screening instruments without compromising performance.

Comparisons across modeling strategies further highlight the importance of how temporal information is incorporated during training. Although all models are evaluated under identical cross-sectional inference conditions, approaches differ in their ability to exploit longitudinal structure. Random forests provide strong nonlinear baselines, while linear models serve as transparent reference points but consistently underperform. Direct neural models trained solely on follow-up predictors achieve competitive performance in several settings, indicating that shared representations across domains can be learned even under limited supervision.

The most consistent gains are achieved through knowledge distillation. By training a compact student network to reproduce the probabilistic outputs of longitudinal teacher models while remaining purely cross-sectional at inference, distillation effectively compresses temporal structure into a deployable screening model. This approach yields systematic improvements over direct neural training and classical baselines across physiological domains and target definitions, particularly for metabolic dysregulation, while maintaining low inference-time complexity.

Beyond improved discrimination, the distilled student architecture offers additional engineering advantages. The low-dimensional latent embedding learned prior to the output heads provides a reusable risk representation that can support late fusion with complementary modalities or downstream screening modules without retraining the full model. This flexibility is difficult to achieve with tree-based or purely linear approaches and positions the student network as a modular component within larger screening or decision-support pipelines.

We deliberately evaluated multiple target constructions—quantile-based, z-score-based, and clinically thresholded—to assess robustness to target operationalization. While some definitions yield slightly higher discrimination in specific domains, performance trends remain consistent across constructions. Treating these targets as complementary operationalizations of latent dysregulation avoids reliance on a single labeling scheme and enhances the generality of the proposed framework.

Several limitations merit consideration. Physiological dysregulation is modeled here as a binary screening outcome, necessarily simplifying continuous biological processes. In addition, although MIDUS provides rich longitudinal data, sample size remains modest relative to predictor dimensionality. Finally, while the proposed models are deployable, prospective clinical impact was not evaluated. These limitations reflect common trade-offs in secondary cohort analyses and motivate future work.

Future work could extend the present framework along several complementary directions, including external validation in independent cohorts, calibration and decision-curve analyses for application-specific screening thresholds, and the incorporation of additional data modalities when available. In particular, the distilled student architecture provides a low-dimensional latent representation that naturally supports late fusion with complementary inputs such as passive sensing signals (e.g., wearable-derived activity or sleep patterns), behavioral markers extracted from speech or movement, or structured electronic health record data. This modular design enables expansion beyond psychometric predictors without retraining the full screening model, preserving deployability while allowing longitudinal and multimodal information to be incrementally integrated. More broadly, the proposed framework offers a general and extensible template for translating high-dimensional longitudinal behavioral data into compact, scalable screening models suitable for population-level deployment.

## 6. Conclusions

We presented a screening-oriented modeling framework for predicting latent physiological dysregulation from high-dimensional psychometric survey data in a longitudinal cohort. By integrating stable feature ranking, parsimony-driven dimensionality reduction, and knowledge distillation, the proposed pipeline transforms rich but highly redundant survey inputs into compact, deployable screening models while preserving longitudinal predictive signal.

Across inflammatory, metabolic, and neuroendocrine domains, we showed that aggressive feature reduction—by more than an order of magnitude—does not degrade screening performance, and that student models trained via distillation consistently exceed the discrimination achieved by direct neural and classical baselines. Longitudinal information is exploited during model development but is not required at inference, enabling scalable deployment without the additional data collection burden.

Beyond improved predictive performance, the distilled student models yield low-dimensional latent representations that support extensibility and modular integration. This property makes the approach particularly well-suited for screening contexts where compactness, stability, and the ability to incorporate additional signals over time are critical. More broadly, the proposed framework provides a principled pathway for translating complex longitudinal cohort data into practical, non-invasive population health screening tools.

## Figures and Tables

**Table 1:** Clinical thresholds used for the clinically thresholded (C) target definition.

Domain	Biomarker	High-Risk Criterion
INFL	CRP	>3.0 mg/L
INFL	IL6	>3.19 pg/mL
INFL	FGN	>400 mg/dL
META	GLU	≥126 mg/dL
META	TG	≥150 mg/dL
META	HDL	<40 (men), <50 (women) mg/dL
META	SBP	≥140 mmHg
META	DBP	≥90 mmHg
META	WC	>102 (men), >88 (women) cm
NEURO	COR	Upper quartile (sex-specific)
NEURO	DHEA	Lower quartile (sex-specific)

**Table 2:** Predictor inclusion and exclusion criteria applied to MIDUS survey variables under the longitudinal screening framework.

Filter Category	Operational Criterion	Rationale
Project scope	Non-survey variables excluded	Restricts predictors to psychometric self-report measures and excludes biomarker, cognitive, and administrative projects.
Canonical biological metrics	Allostatic load components and related variables excluded	Prevents indirect reuse of biomarker-derived information in the predictor set.
Longitudinal consistency	Variables lacking valid MIDUS 2 and MIDUS 3 counterparts excluded	Ensures predictor availability across waves and supports longitudinal screening.
Target circularity	Variables used in physiological target construction (and cross-wave counterparts) excluded	Preserves biological independence between predictors and targets.
Administrative and linkage fields	Identifiers, linkage variables, weights, and process metadata excluded	Removes non-informative variables unsuitable for modeling or deployment.
Redundant questionnaire variants	Alternate or duplicated scale versions collapsed or removed	Enforces canonical questionnaire representations and reduces redundancy.
Data usability	Variables with no observed values or zero variance after numeric coercion excluded	Ensures all retained predictors contribute usable information.

**Table 3:** Training hyperparameters used for teacher and student models.

Component	Hyperparameter	Value
Teacher model (random forest)	Number of trees	200
	Class weighting	balanced
	Random seed	42
Student model (MLP)	Hidden layers	2
	Hidden width	128 neurons
	Embedding dimension	32
	Activation function	ReLU
	Dropout	0.25
	Output heads	3
	Optimizer	AdamW
	Learning rate	$1\times 10^{-3}$
	Weight decay	$1\times 10^{-4}$
	Batch size	64
	Maximum epochs	200
	Early stopping patience	20
	Distillation loss	BCE + MSE
	Distillation mixing coefficient (α)	0.7

**Table 4:** Cohort size, candidate predictor dimensionality, and follow-up (MIDUS 3) positive class counts for each physiological domain under each target definition. Positives correspond to individuals labeled as high risk at follow-up.

Target Definition	INFL Positives	META Positives	NEURO Positives
Quantile (Q)	255	275	265
Clinical (C)	129	235	265
Z-score (Z)	146	146	146

Note: The analytic cohort size was n=584 for all experiments, and the post-filter candidate predictor universe contained p=1904 cross-wave survey variables.

**Table 5:** Outcome-stage (MIDUS 3) screening performance of longitudinal random forest teacher models trained on the full predictor set (FULL) and the parsimony-optimal subset ($K^{*}$). Performance is reported as out-of-fold AUC and average precision (AP), with boldface denoting the higher-performing model for each metric.

Definition	Predictors	INFL		META		NEURO	
		**AUC**	**AP**	**AUC**	**AP**	**AUC**	**AP**
Quantile (Q)	FULL (=1904)	0.644	0.574	0.735	0.720	0.612	0.540
	$K^{*}=50$	**0.675**	**0.600**	**0.754**	**0.732**	**0.660**	**0.595**
Clinical (C)	FULL (=1904)	0.681	0.379	0.752	0.627	0.612	0.540
	$K^{*}=65$	**0.689**	**0.381**	**0.748**	**0.632**	**0.631**	**0.577**
Z-score (Z)	FULL (=1904)	0.697	0.459	0.625	0.346	0.523	0.259
	$K^{*}=55$	**0.703**	**0.455**	**0.689**	**0.415**	**0.574**	**0.286**

**Table 6:** A comparison of out-of-fold screening performance on parsimony-optimal predictor sets ($K^{*}$) across all target definitions. Performance is reported as AUC and average precision (AP) for inflammatory (INFL), metabolic (META), and neuroendocrine (NEURO) dysregulation at follow-up, with boldface denoting the higher-performing model for each metric.

Target	Model	INFL		META		NEURO	
		**AUC**	**AP**	**AUC**	**AP**	**AUC**	**AP**
Quantile (Q)	RF (M3-only)	0.679	0.612	0.753	0.738	0.644	0.581
	Logit (L2)	0.635	0.581	0.655	0.605	0.624	0.569
	Elastic Net	0.640	0.587	0.662	0.614	0.624	0.562
	MLP (direct)	0.674	0.589	0.729	0.708	0.626	0.568
	MLP (distill)	**0.714**	**0.669**	**0.775**	**0.774**	**0.676**	**0.615**
Clinical (C)	RF (M3-only)	0.688	0.358	0.749	0.633	0.626	0.577
	Logit (L2)	0.679	0.390	0.650	0.532	0.585	0.537
	Elastic Net	0.683	0.398	0.654	0.534	0.587	0.531
	MLP (direct)	0.616	0.305	0.679	0.561	0.589	0.521
	MLP (distill)	**0.723**	**0.469**	**0.761**	**0.680**	**0.677**	**0.608**
Z-score (Z)	RF (M3-only)	0.702	0.432	0.676	0.427	0.557	0.283
	Logit (L2)	0.670	0.411	0.581	0.321	0.548	0.273
	Elastic Net	0.674	0.416	0.582	0.320	0.553	0.275
	MLP (direct)	0.672	0.375	0.645	0.379	0.556	0.296
	MLP (distill)	**0.745**	**0.519**	**0.718**	**0.481**	**0.669**	**0.423**

**Table 7:** Average precision (AP) and AP lift for the distilled student model (MLP distill), reported relative to class prevalence. AP lift is defined as AP/π, where π denotes the positive class rate.

Target	Domain	Prevalence π	AP	AP Lift
Quantile (Q)	INFL	0.437	0.669	1.53
Quantile (Q)	META	0.471	0.774	1.64
Quantile (Q)	NEURO	0.454	0.615	1.36
Clinical (C)	INFL	0.221	0.469	2.12
Clinical (C)	META	0.402	0.680	1.69
Clinical (C)	NEURO	0.454	0.608	1.34
Z-score (Z)	INFL	0.250	0.519	2.08
Z-score (Z)	META	0.250	0.481	1.92
Z-score (Z)	NEURO	0.250	0.423	1.69

**Table 8:** Top predictors of physiological dysregulation identified by the feature selection pipeline. Mean and standard deviation of permutation importance (Δ AUC) are computed across nine model variants corresponding to three dysregulation definitions (Q, C, Z) and three physiological subsystems (I = inflammatory, M = metabolic, N = neuroendocrine).

Construct	Domain	Mean	Std	Q	C	Z
		Δ **AUC**	Δ **AUC**	**Models**	**Models**	**Models**
Body mass index	Physical Health	0.0155	0.0153	I,M,N	I,M	I,M,N
Waist around navel (inches)	Physical Health	0.0082	0.0075	I,M,N	I,M	I,M,N
Year of last menstrual period (irregular)	Physical Health	0.0064	0.0106	–	–	M,N
Current worth of respondent’s IRA account	Socioeconomic	0.0063	0.0086	–	I,M,N	–
Hips at widest point (inches)	Physical Health	0.0040	0.0053	I,N	I,M,N	I,N
How often at least one drink (past month)	Health Behaviors	0.0038	0.0037	I,M,N	–	–
Years lived in this state	Geography & Context	0.0033	0.0061	I,M,N	M,N	N
Respondent’s wages last calendar year	Socioeconomic	0.0028	0.0054	–	–	I,N
Weight current (pounds)	Physical Health	0.0028	0.0048	M	M,N	I,M,N
Number years ago told high blood pressure	Physical Health	0.0027	0.0059	M	I,M,N	M,N
Number years employed ≥6 months/year	Socioeconomic	0.0026	0.0064	N	I,M,N	N
State most like to live next 10 years	Geography & Context	0.0025	0.0035	I,M,N	M,N	I,M,N
Positive relations with others (PWB 7-item)	Psychological & Social	0.0024	0.0042	I,N	–	I,N
Age feel like most of the time	Psychological & Social	0.0024	0.0027	I,M,N	I,N	–
High blood pressure ever diagnosed	Physical Health	0.0023	0.0053	–	–	M,N

**Table 9:** Distribution of retained predictors by conceptual domain.

Domain	Number of Predictors	Percentage
Psychological & Social	26	33.77%
Physical Health	17	22.08%
Socioeconomic	15	19.48%
Demographics	12	15.58%
Health Behaviors	4	5.19%
Geography & Context	3	3.90%

**Table 10:** Positioning of the present study relative to the representative literature examining psychosocial determinants of physiological dysregulation and behavioral health prediction.

Study	Dataset	Objective	Method	Key Finding
Brooks et al. (2014) [22]	MIDUS	Social relationships and allostatic load	Regression	Relationship strain associated with higher allostatic load
Lewis & Hill (2023) [23]	HRS, ELSA	Purpose in life and physiological regulation	Longitudinal regression	Higher purpose linked to lower dysregulation
Surachman et al. (2023) [21]	MIDUS Refresher	Financial hardship and inflammation	Regression	Financial stress associated with inflammatory biomarkers
Miotto et al. (2016) [8]	EHR datasets	Disease risk prediction	Deep learning	Latent representations enable predictive risk stratification
This study	MIDUS	Screening physiological dysregulation	Distilled neural network	Psychometric predictors enable non-invasive screening

## Data Availability

The data presented in this study are available from the Midlife in the United States (MIDUS) study and are publicly accessible through the Inter-university Consortium for Political and Social Research (ICPSR). Specifically, we used data from MIDUS Wave 2 (2004–2006) and Wave 3 (2013–2014) Survey Projects, as well as the corresponding MIDUS Biomarker Projects. Access to these datasets requires registration with ICPSR and acceptance of the MIDUS data use agreement. The datasets can be obtained from https://www.icpsr.umich.edu. The data are not redistributed with this article. Derived data, analytic code, and scripts supporting the findings of this study are available from the corresponding author upon reasonable request.
